# Human epidermal growth factor 2 overexpressed alpha-fetoprotein-producing-gastric cancer

**DOI:** 10.1007/s12672-023-00731-1

**Published:** 2023-06-24

**Authors:** Hiroko Shimizu, Mitsugu Kochi, Masashi Fujii, Megumu Watabe, Yoritaka Matsuno, Takaharu Kawai, Hiroshi Suda, Tomoyuki Tanino, Yoko Nakanishi, Shinobu Masuda, Yukiyasu Okamura

**Affiliations:** 1 Division of Digestive Surgery, Department of Surgery, Nihon University School of Medicine, Tokyo, Japan; 2 Japan Clinical Cancer Research Organization, Tokyo, Japan; 3 Division of Oncologic Pathology, Department of Pathology and Microbiology, Nihon University School of Medicine, Tokyo, Japan; 4 Division of Oncologic Pathology, Department of Pathology and Microbiology, Nihon University School of Medicine, 30-1 Ohyaguchi-Kamicho, Itabashi-ku, Tokyo, 173-8610 Japan

**Keywords:** AFP-producing gastric cancer, HER2, Mixed histological subtype

## Abstract

**Purpose:**

This study aimed to elucidate the clinicopathological characteristics of α-fetoprotein (AFP)-producing gastric carcinoma (AFP-GC) with human epidermal growth factor receptor (HER)2 overexpression to extend the treatment strategy for AFP-GC.

**Methods:**

We analyzed 41 patients with AFP-GC who underwent surgical resection or chemotherapy from 1989 to 2019, and who had over 20ng/mL of serum AFP or positive immunohistochemical AFP expression. HER2 expression status was investigated by immunohistochemistry (IHC) for all patients and by fluorescence in situ hybridization (FISH) for cases with an IHC score of 2+. AFP-GC with an IHC score of 3 + or 2 + and FISH positivity was defined as HER2 overexpressed AFP-GC. The correlation between HER2 status and clinicopathological characteristics and prognosis in AFP-GC was analyzed.

**Results:**

HER2 overexpression was detected in 17.1% of AFP-GC patients. The prognosis of the patients with HER2 overexpressed AFP-GC was not significantly different compared to HER2 non-overexpressed AFP-GC. HER2 overexpressed AFP-GC consisted of heterogeneous histology with a higher proportion of mixed-type tumors (p = 0.002). The clinical outcome of AFP-GC with mixed-type of histology tended to be better than other intestinal or diffuse types (p = 0.05).

**Conclusion:**

HER2 overexpressed AFP-GC consisted of a mixed type of histology, which showed a better prognosis. The results presented that HER2 status in AFP-GC is one of the molecular candidates to improve the prognosis.

## Introduction

Alpha-fetoprotein (AFP)-producing gastric cancer (AFP-GC) was first reported by Bourreille et al. in 1970 [1]. Alpha-fetoprotein is a glycoprotein produced in fetal hepatocytes and yolk sacs [2]. It is rarely produced in the tissues of healthy adults but is produced by tumor cells such as hepatocellular carcinoma, hepatoblastoma, and York-Sack tumor, and, rarely, by lung and gastric cancer. AFP-GC has subsequently been followed by many reports [3–6]. Studies have shown that liver metastases frequently occur as a pathophysiology of AFP-GC [6–10], and even if the tumor is diagnosed in the early phase, the prognosis is poor [9–16]. To date, there is still much controversy regarding AFP-GC treatment.

Many studies have shown that human epidermal growth factor receptor (HER) 2 overexpression is detected in 7–34% of gastric cancer cases [17–25]. The effectiveness of trastuzumab and lapatinib has been demonstrated in different gastric cancer models and has led to clinical studies. Trastuzumab, a monoclonal antibody against HER2 (also known as ERBB2), in combination with chemotherapy is considered a new standard for patients with advanced gastric or gastroesophageal junction cancer with HER2 overexpression [25]. In AFP-GC, however, the population of HER2 overexpressed tumors and their clinicopathological characteristics are still unclear.

This study aimed to clarify the population of HER2 overexpressed AFP-GC and their clinicopathological characteristics, in real-world patients, to extend the treatment strategy for AFP-GC using anti-HER2 agents.

## Materials and methods

### Patients

From January 1989 to December 2019, stomach adenocarcinoma patients who had been diagnosed and undergone surgical resection or chemotherapy at Nihon University Itabashi Hospital were retrospectively reviewed. Formalin-fixed and paraffin-embedded (FFPE) tissue specimens of the primary region without any anti-cancer treatment were obtained by resection or biopsy. We analyzed 41 patients who had over 20ng/mL of serum AFP or whose FFPE tissue sections were positive immunohistochemical AFP expression. Serum AFP levels were determined using a commercial enzyme immunoassay kit (Fujirebio Inc., Tokyo, Japan), and a cut-off value of 20 ng/mL were used. Tumor AFP expression was analyzed by immunohistochemistry (IHC) using primary antibodies against AFP (IR500, rabbit polyclonal, Agilent Technologies, Santa Clara, CA, USA) and Simple Stain MAX-PO (Multi) (Nichirei Bioscience Inc., Tokyo, Japan). AFP expression was evaluated as positive when > 5% of tumor cells were stained. The summary of the patients is shown in Table 1. All 41 patients were not treated with trastuzumab. All procedures in our study were performed following the ethical standards of the institutional and national research committees, and the Declaration of Helsinki. This study was approved by the institutional review board of Nihon University Itabashi Hospital (RK-150609-07).

**Table 1:** Summary of the patients

Clinicopathological factors	Total
	N = 41
Sex	
Male	31 (75.6)
Female	10 (24.4)
Age (Year)	
Median [Range]	69 [52–81]
52–59	8 (19.5)
60–69	14 (34.1)
70–79	17 (41.5)
80–81	2 (4.9)
Location	
Upper	15 (36.6)
Middle	16 (39.0)
Lower	10 (24.4)
Clinical Stage	
Stage I-III	16 (39.0)
Stage IV	25 (61.0)
Operation	
Non-operated	16 (39.0)
Operated	25 (61.0)
Histologic subtype^†^	
Intestinal	17 (41.5)
Diffuse	10 (24.4)
Mixed	14 (34.1)
Serum AFP level (ng/mL)	
Median [Range]	605 [7.7-273000]
7.7–19.9 (normal)	3 (7.3)
20.0-99.9	10 (24.4)
100–499	3 (7.3)
500–999	8 (19.5)
1000–9999	12 (29.3)
10,000–273,000	5 (12.2)

†Lauren’s classification

### Immunohistochemistry for HER2

To investigate immunohistochemical HER2 expression status, FFPE tissue specimens of 41 patients were cut into 4-µm-thick sections and mounted on silane-coated glass slides. After deparaffinization, HER2 expression was analyzed using HercepTest (Agilent Technologies, Santa Clara, CA, USA) according to the manufacturer’s instructions. HER2 expression was evaluated by certified board pathologists according to the scoring system by Hofmann et al. [26] and the 2018 ASCO/CAP guidelines [27] as follows. Positive; strongly positive and completely membranous staining (3+) in ≥ 10% of tumor cells. Equivocal; moderately positive staining for complete membranous staining (+ 2) in ≥ 10% of tumor cells. Negative; no reactivity or membranous staining in < 10% of tumor cells, or faint and partial membrane reactivity (1+) in ≥ 10% of tumor cells [26, 27].

### Fluorescence in situ hybridization for HER2

When the HER2 expression of the tested samples was evaluated as equivocal by IHC, we determined whether HER2 DNA was amplified by fluorescence in situ hybridization (FISH) methods using in vitro diagnostics (IVD) kit Histra HER2 FISH (JOKOH CO., LTD., Tokyo, Japan). FFPE tissue specimens were cut into 4-µm-thick sections and mounted on silane-coated glass slides. After deparaffinization, FISH analysis was performed according to the manufacturer’s instructions. Fluorescence signals of HER2 and CEP17 were acquired with an Axio Imager Z2 Upright Microscope (Carl Zeiss, Oberkochen, Germany) and ZEN 2 pro software (Carl Zeiss). The HER2 DNA amplification was determined when the signal counts of HER2/CEP17 were ≥ 2.0.

### Statistical analysis

The association between HER2 status and clinical and clinicopathological factors was evaluated using the chi-squared test. Survival assays were performed using the Cox proportional hazards and Kaplan–Meier models.

Significance was set at p < 0.05. The SAS software package for Windows, version 8.02 (SAS Institute Inc., Cary, NC, USA) and Microsoft Excel 2016 (Microsoft Co., Ltd., Japan) were used for statistical analysis and data calculation.

## Results

### Her2 overexpression in AFP-GC

**Fig. 1:**
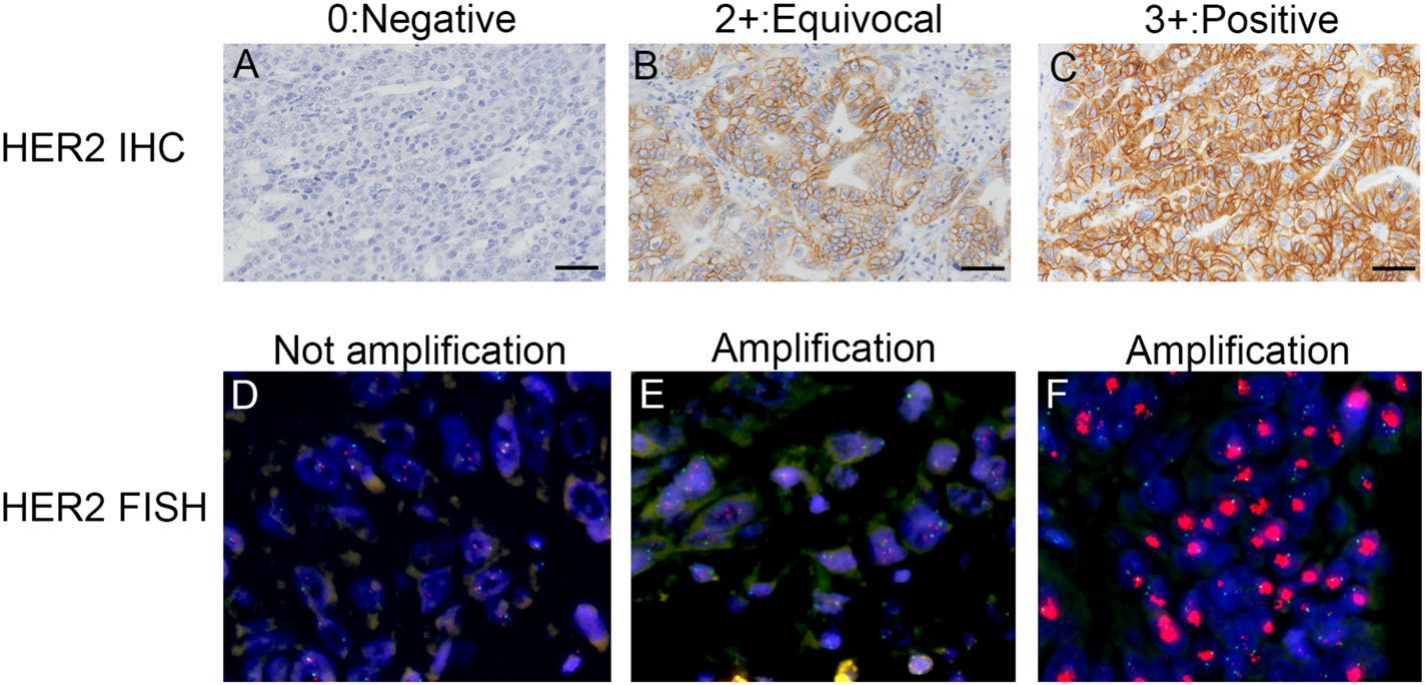
HER2 status by IHC and FISH. **A** Negative expression (0), **B** equivocal expression (2+), and **C** positive expression (3+) by immunohistochemistry. Each bar shows 50 μm. Equivocal samples need additional FISH analysis to determine whether their HER2 DNA was no amplification **D** or amplification **E**, **F**. The green signals show CEP17 and the red signals show HER2. The HER2 DNA amplification was determined when the signal counts of HER2/CEP17 were ≥ 2.0 in 20 tumor cells

The photos of HER2 overexpression by IHC and HER2 amplification by FISH are shown in Fig. 1. Table 2 shows that HER2 overexpression was detected in seven (17.1%) of the 41 AFP-GC patients. The breakdown was 3 + of HER2 score was detected in five (12.2%) patients, and 2 + with gene amplification confirmed by FISH was observed in two (4.9%) patients.

**Table 2:** HER2 status assessed by IHC and FISH

HER2 status	Number	%
Positive		
IHC 3+	5	12.2
IHC 2+ and FISH positive	2	4.9
Total	7	17.1
Negative		
IHC 0, 1+	31	75.6
IHC 2+ and FISH negative	3	7.3
Total	34	82.9

### Clinicopathological features of HER2 overexpressed AFP-GC

**Fig. 2:**
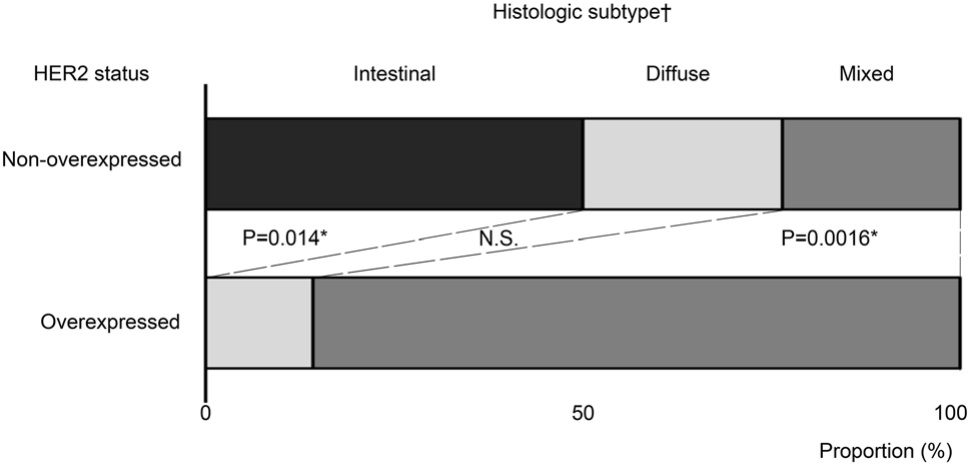
The proportion of histologic subtype by Lauren’s classification in the HER2 overexpressed and the HER2 non-overexpressed AFP-GC groups †, Lauren’s classification. ** P < 0.01, * P < 0.05, by residue analysis

We compared the clinicopathological features of HER2 overexpressed patients to non-overexpressed patients within AFP-GC. Table 3 shows the correlation between clinicopathological features and HER2 status in AFP-GC, and that the proportion of histologic subtypes evaluated by Lauren’s classification [28] was significantly different between the HER2 overexpressed group and the non-overexpressed group (p = 0.005). By additional residue analysis, Fig. 2 shows that mixed histology was detected at a significantly higher proportion in the HER2 overexpressed group (p = 0.002), and intestinal histology was significantly higher in the HER2 non-overexpressed group (p = 0.01). Other factors, including sex, age, tumor location, operation, clinical stage, and serum AFP level, were not significantly different between HER2 overexpressed AFP-GC and HER2 non-overexpressed AFP-GC are shown in Table 3.

**Table 3:** Correlation between HER2 status and clinicopathological features in AFP-GC

Clinicopathological factors	Total	HER2 status		P value
		Overexpression	Non-overexpression	
	N = 41	N = 7 (16.6%)	N = 34 (83.3%)	
Sex				
Male	31	5 (71.4)	26 (76.5)	0.78
Female	10	2 (28.6)	8 (23.5)	
Age (Year)				
<70	22	4 (57.1)	18 (52.9)	0.59
≥70	19	3 (42.9)	16 (47.1)	
Location				
Upper	15	3 (42.9)	12 (35.3)	0.71
Middle	16	4 (57.1)	12 (35.3)	
Lower	10	0 (0.0)	10 (29.4)	
Clinical stage				
Stage I-III	16	3 (42.9)	13 (38.2)	0.82
Stage IV	25	4 (57.1)	21 (61.8)	
Operation				
Non-operated	16	2 (28.6)	14 (41.2)	0.53
Operated	25	5 (71.4)	20 (58.8)	
Histologic subtype^†^				
Intestinal	17	0 (0.0)	17 (50)	0.005*
Diffuse	10	1 (14.3)	9 (26.5)	
Mixed	14	6 (85.7)	8 (23.5)	
Serum AFP level (ng/mL)				
< 500	16	3 (42.9)	13 (38.2)	0.57
≥ 500	25	4 (57.1)	21 (61.8)	

^†^Lauren’s classification, *P < 0.01, chi-square test

**Fig. 3:**
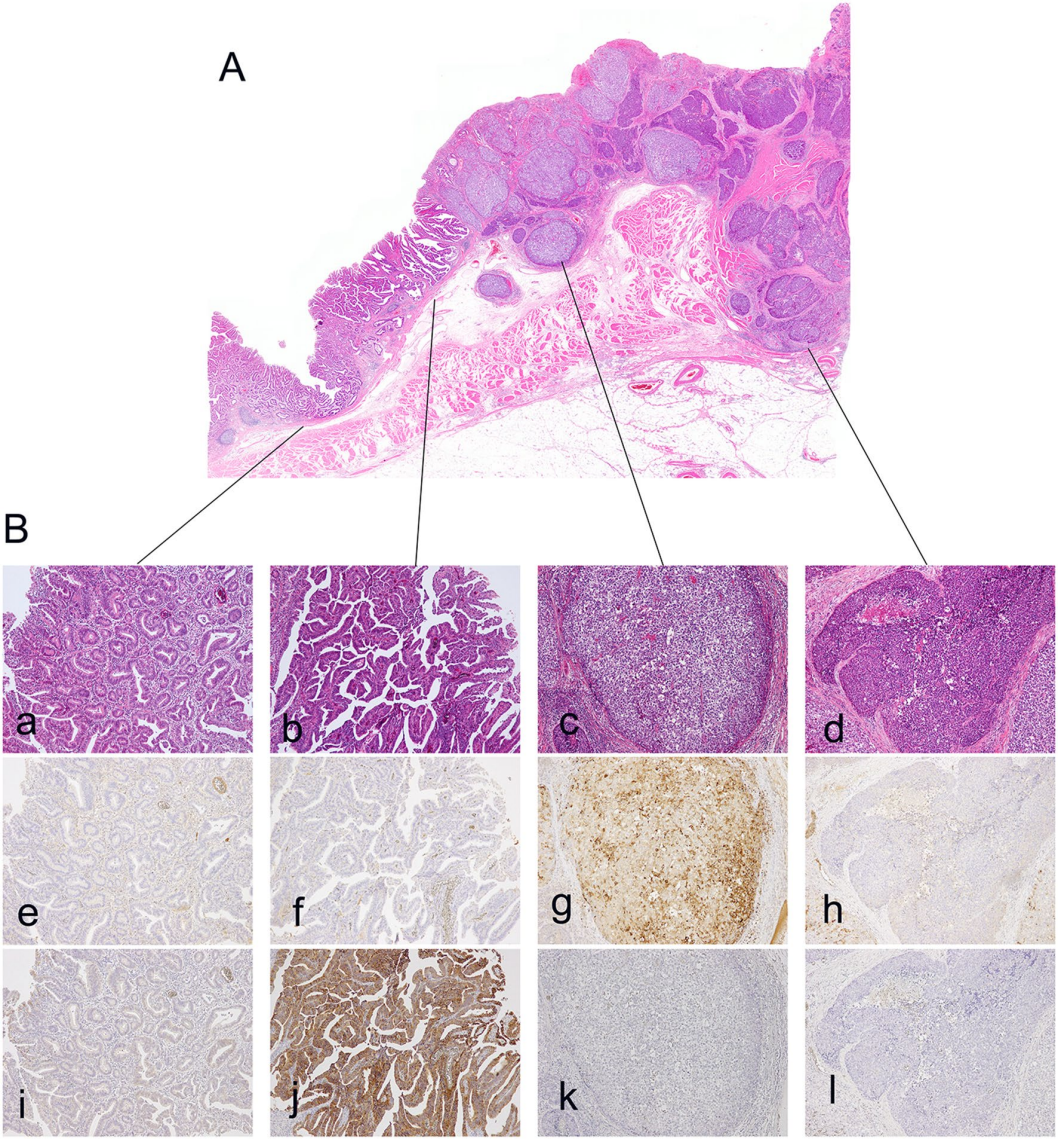
Histopathological findings of a representative case of mixed type of histology. Whole H&E stained tissue section **A** and detailed structures **B**. This gastric tumor consists of heterogeneous cancer cells with different structural features: tubular (a, e, i), papillary (b, f, j), hepatoid (c, g, k), and solid (d, h, l) patterns. The tumor was diagnosed as a mixed type when both intestinal and diffuse types were detected. Each component showed a different immunohistochemical phenotype for AFP (e, f, g, h) and HER2 (i, j, k, l)

Histological findings of a representative case of mixed type of histology are shown in Fig. 3. Figure 3A shows H&E stained whole tissue section. Figure 3B shows the details of mixed histology types contained in a single tissue section. In this tumor, different tubular, as shown in Fig. 3B-a, e, i, papillary, as shown in Fig. 3B-b, f, j, hepatoid, as shown in Fig. 3B-c, g, k, and solid, as shown in Fig. 3B-d, h, l, pattern structures consisted. Each component showed a different immunohistochemical phenotype for AFP (Fig. 3B-e, f, g, h) and HER2 (Fig. 3B-I, j, k, l).

### Relationship between HER2 overexpression and patients’ prognosis in AFP-GC

We next investigated the prognostic implication of HER2 expression in AFP-GC patients, compared to other clinicopathological factors. Survival analysis was performed by classifying the present AFP-GC patients into two groups according to the clinicopathological factors as follows; HER2 overexpressed and non-overexpressed groups, female and male, < 70 years and ≥ 70years, lower and upper/middle of tumor location, clinical stage I – III and stage IV, operated and non-operated, < 500ng/ml and ≥ 500ng/ml of serum AFP level, and intestinal/diffuse type and mixed type of histologic subtype classified by Lauren’s classification.

**Fig. 4:**
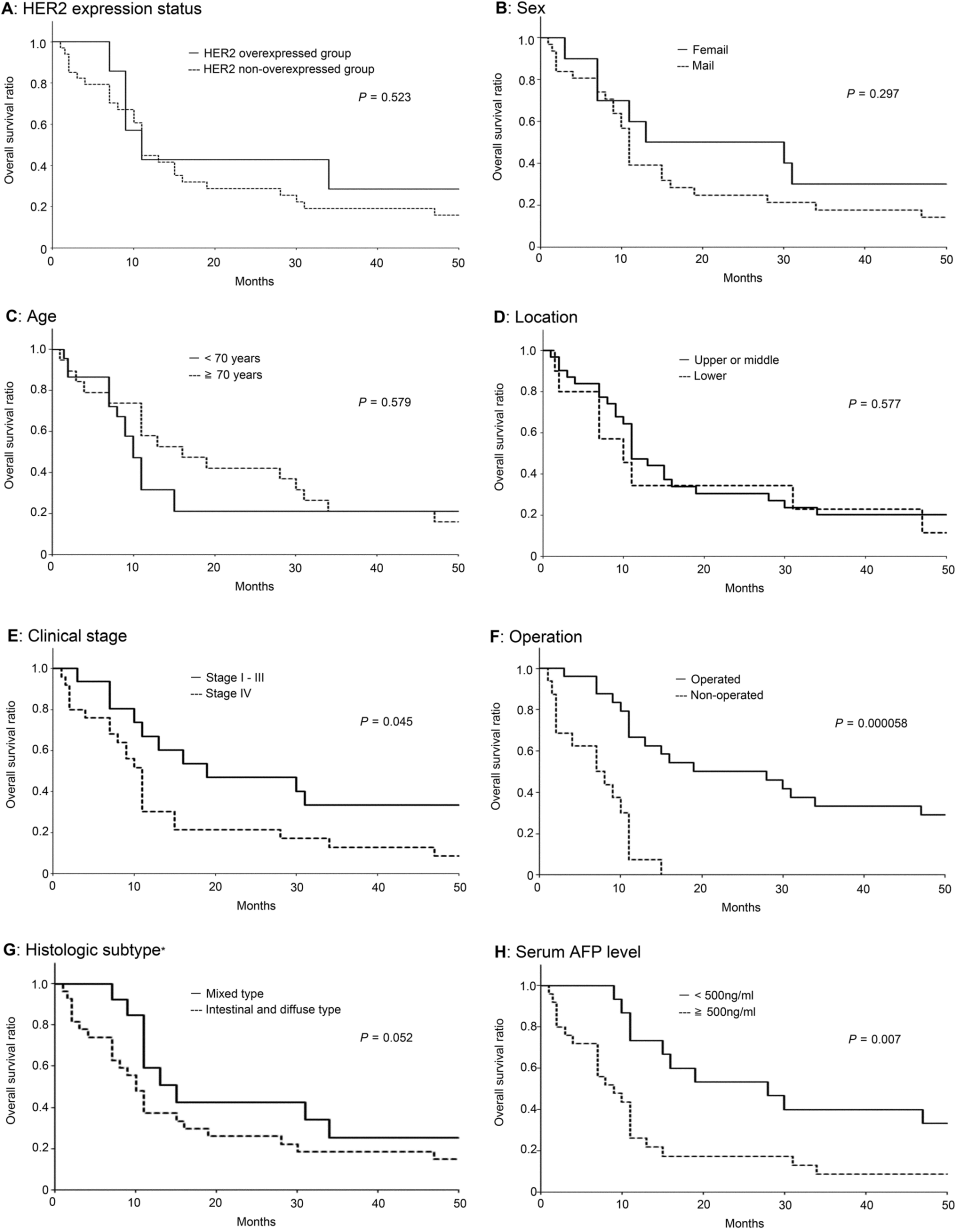
Overall survival of AFP-GC. Overall survival of HER2 overexpressed and non-overexpressed groups **A**, female and male **B**, < 70 years and ≥ 70years **C**, lower and upper/middle of tumor location **D**, clinical stage I – III and stage IV **E**, operated and non-operated **F**, intestinal/diffuse type and mixed type of histology classified by Lauren’s classification **G**, and < 500ng/ml and ≥ 500ng/ml of serum AFP level **H**, analyzed using the Kaplan–Meier methods (log-rank), are shown

Figure 4A, H show the results of the Kaplan-Meyer analysis that showed the differences in the overall survival rate of each AFP-GC group. Overall survival in HER2 overexpressed group and HER2 non-overexpressed group was not significantly different (p = 0.52, log-rank) as shown in Fig. 4A. Patients with clinical stage IV showed significantly worse prognosis than those with stage I - III (p = 0.045) as shown in Fig. 4E. Operated patients had a significantly better prognosis than non-operated patients (p < 0.001) as shown in Fig. 4F. Patients with ≥ 500ng/mL of serum AFP levels had a significantly worse prognosis than those with < 500ng/mL (p = 0.007) as shown in Fig. 4H. Patients with mixed histology tended to have a better prognosis than those with intestinal or diffuse histology (p = 0.05) as shown in Fig. 4G. Other clinicopathological factors, sex, age, and tumor location status were not significantly correlated with clinical outcomes as shown in Fig. 4B C, and D, respectively.

Multivariate survival analysis was performed by Cox proportional hazards model. These results are shown in Table 4. The one- and 3-year survival rates were 42.9% and 28.6% in the HER2 overexpressed group, and 46.7% and 17.6% in the HER2 non-overexpressed group. The hazards of the HER2 overexpressed group were 0.95 times those of the HER2 non-overexpressed group, and 95% confidence interval (CI) was 0.26–3.48 (p = 0.94).

**Table 4:** Multivariate survival analysis by Cox proportional hazards model

Covariate	1-year survival rate (%)	3-year survival rate (%)	HR	95% CI	P value
Sex					
Female	60.0	30.0			
Male	35.5	16.1	1.94	0.64–5.94	0.24
Age					
< 70 years	57.9	21.1			
≥ 70 years	27.3	18.2	1.55	0.64–3.73	0.33
Location					
Upper or middle	46.7	20.0			
Lower	30.0	20.0	1.35	0.54–3.37	0.52
Clinical stage					
Stage I-III	66.7	33.3			
Stage IV	29.2	12.5	1.21	0.39–3.76	0.74
Operation					
Non-operated	6.3	0.0			
Operated	64.0	32.0	0.24	0.078–0.73	0.01*
Serum AFP level					
< 500ng/ml	68.8	37.5			
≥ 500ng/ml	24.0	8.0	3.20	1.04–9.82	0.04*
Histologic subtype^†^					
Intestinal or Diffuse type	37.0	18.5			
Mixed type	64.3	35.7	0.42	0.15–1.20	0.11
HER2 status					
Non-overexpressed	46.7	17.6			
Overexpressed	42.9	28.6	0.95	0.26–3.48	0.94

^†^Lauren’s classification; *P < 0.05; HR, hazard ratio; CI, confidential interval

In comparison by other clinicopathological factors, the one- and 3-year survival rates were 24.0% and 8.0% in the patients with ≥ 500ng/mL of serum AFP levels, and 68.8 and 37.5% in those with < 500ng/mL of serum AFP levels (HR, 3.20; 95%CI 1.04–9.82; p = 0.04). The one- and 3-year survival rates were 64.0% and 32.0% in the operated group, and 6.3% and 0.0% in the non-operated group (HR, 0.24; 95%CI 0.078–0.73; p = 0.01). In the patients with mixed histology, the one- and 3-year survival rates were 64.3% and 35.7%, and 37.0% and 18.5% in those with intestinal or diffuse histology (HR, 0.42; 95%CI 0.15–1.20; p = 0.11). Other factors, age, sex, clinical stage, and tumor location, were also not significant.

## Discussion

Even if AFP-GC is diagnosed in an earlier phase, its prognosis is poor due to the susceptibility to liver metastasis and the lower radical resectability [6–10], and no treatment strategy has been established as yet. In contrast, HER2 overexpressed gastric cancer has been established in clinical trials. The ToGA trial was the first phase III trial to add trastuzumab to standard chemotherapy and included patients with HER2-overexpressing advanced gastric or gastroesophageal junction cancer who were randomized to receive 5-fluorouracil/capecitabine and cisplatin alone or in combination with trastuzumab. The results demonstrated the efficacy of HER2-targeted molecular therapy for gastric cancer [25].

Both AFP-GC and HER2 overexpressed gastric cancer has been known for aggressive clinical behavior and poor prognosis. However, no study has addressed HER2 overexpression in AFP-GC. This study revealed the incidence of HER2 overexpression in 17.1% of AFP-GC. The percentage is within the scope of the frequency of all gastric cancer [17–25]. It was also revealed that the prognosis of HER2 overexpressed AFP-GC was not worse than HER2 non-overexpressed AFP-GC, contrasted to the worse prognosis of the only HER2 overexpressed gastric cancer that had been reported [21, 24]. All patients in this study were not treated with trastuzumab, therefore, we don’t have to consider the influence of trastuzumab on the prognosis for HER2 overexpressed AFP-GC in this study. Instead, HER2 overexpressed AFP-GC consisted of a mixed type of histology, and its clinical outcome tended to be better. These results suggested that HER2 overexpressed cancer cells may occur in AFP-GC, consisting of heterogeneous subtypes with a better clinical outcome, compared with HER2 non-overexpressed AFP-GC, which consists of homologous cancer cells with aggressive clinical behavior. To address the relationship between HER2 expression status and the therapeutic effects including trastuzumab and prognosis, a larger number of cohorts are required to study in the future. The cancer genome atlas [29] categorized gastric cancers as Epstein–Barr virus-positive, microsatellite instability, genomic instability, and chromosomal instability (CIN). AFP-GC and HER2 overexpressed gastric cancer were categorized into the CIN subtype, which was the largest category, comprising approximately 50% of gastric cancers. The present study showed that the CIN category has the potential to be sub-grouped according to AFP and/or HER2 overexpression.

Several AFP-producing gastric cancers have been reported to be successfully treated with combined neoadjuvant chemotherapy with epirubicin (EPI), 5-fluorouracil (5-FU), and leucovorin (LV) [30]. We performed combination chemotherapy with 5-FU, LV, etoposide (VP-16), and cis-diamminedichloroplatinum (CDDP) specified by Nakajima et al. as the FLEP regimen for patients with stage IV gastric cancer who were not candidates for surgery [31]. The purpose of the FLEP therapy, which consisted of a combination of local delivery of VP-16 and CDDP to the aorta and systemic delivery of 5-FU and LV, was the control of both local and disseminated disease in the intra- and extra-abdominal regions. In our previous observational study of FLEP chemotherapy, the median survival time in the group with AFP-GC was 15.8 months compared to 10.3 months in the non-AFP-GC group. The cumulative survival of stage IV patients with AFP-GC was significantly higher than those with non-AFP-GC [16]. This finding has suggested that AFP-GC has high chemosensitivity. Based on our results, heterogeneity of cancer cells with different susceptibility or resistance to chemotherapy may affect the prognosis. Furthermore, evidence for novel therapies targeting HER2 or AFP molecules has accumulated. For example, lapatinib was shown to be more effective for HER2-positive AFP-GC [15, 32], and ramucirumab was reported to be more effective for AFP-positive hepatocellular carcinoma (HCC) than for AFP-negative HCC [33].

The clinical aggressiveness of HER2 non-overexpressed AFP-GC may also apply to other new anticancer medicines. Genetic testing is becoming more widely available for various carcinomas [34]. Currently, the AFP-GC needs to select the appropriate drug using a panel test. Some investigators have reported that the higher expression of c-Met may explain the poorer prognosis of AFP-GC [35]. When the different biochemical mechanisms and oncogenes of AFP-GC are revealed, it may be possible to use this information in the therapeutic management of this cancer.

In conclusion, HER2 overexpression was detected in 17.1% of AFP-GC. HER2 overexpressed AFP-GC consisted of a mixed type of histology, which showed a better prognosis. These results showed that HER2 status in patients with AFP-GC should be examined to investigate its unique characteristics from clinical, pathological, and molecular aspects and improve the prognosis of patients with AFP-GC by providing optional treatments for molecular targets.

## Data Availability

The authors confirm that the data supporting the findings of this study are available within the article.
